# Risk of Postoperative Hemorrhage After Glioma Surgery in Patients with Preoperative Acetylsalicylic Acid

**DOI:** 10.3390/cancers16223845

**Published:** 2024-11-15

**Authors:** Anatoli Pinchuk, Nikolay Tonchev, Claudia A. Dumitru, Belal Neyazi, Klaus-Peter Stein, I. Erol Sandalcioglu, Ali Rashidi

**Affiliations:** Department of Neurosurgery, Otto-von-Guericke-University Magdeburg, 39120 Magdeburg, Germany; anatoli.pinchuk@med.ovgu.de (A.P.); nikolay.tonchev@med.ovgu.de (N.T.); belal.neyazi@med.ovgu.de (B.N.); klaus-peter.stein@med.ovgu.de (K.-P.S.); erol.sandalcioglu@med.ovgu.de (I.E.S.)

**Keywords:** acetylsalicylic acid, glioma, craniotomy, postoperative hemorrhage

## Abstract

Background/Objectives: Patients with gliomas show an increased risk of spontaneous hemorrhages throughout the disease. Simultaneously, the number of patients taking acetylsalicylic acid (ASA) for primary and secondary prophylaxis is rising in daily clinical practice, and interrupting ASA intake before elective or emergency intracranial surgery is not always feasible. This study aims to evaluate the risks associated with continuing ASA use perioperatively while focusing on hemorrhage and potential thromboembolic events that may arise from discontinuing ASA, particularly in multimorbid patients undergoing glioma surgery. Methods: The clinical parameters and imaging data of 7149 patients who underwent intracranial surgery in our department over a 10-year period were retrospectively analyzed. Patients were categorized into two groups based on their ASA status: Group 1 (no ASA impact) included those with no ASA use or who discontinued ASA use more than seven days prior to surgery (low stroke or cardiovascular risk), and Group 2 (ASA impact) included those who continued ASA use within seven days prior to operation (high stroke or cardiovascular risk). Results: In this retrospective study, data from 650 patients with various types of glial tumors who underwent surgery between 2008 and 2018 were examined. Of these patients, 50 experienced a postoperative hemorrhage (POH), and 10 required reoperations due to clinical neurological deterioration and increased intracranial pressure caused by the space-occupying effect of the hemorrhage. In the ASA impact group, 2.7% developed POH, compared to 1.3% in the no ASA impact group (*p* = 0.098). Our analysis did not show a significantly increased risk of POH after surgery, although patients in the ASA impact group had a one- to two-fold higher risk of developing POH overall. Additionally, other factors contributing to postoperative hemorrhage following glioma surgery were investigated and evaluated. Conclusions: In this cohort, the perioperative use of ASA was not associated with an increased rate of hemorrhagic complications after intracranial glioma surgery, although a trend was observed. In patients with high stroke and cardiovascular risk, ASA can be continued during elective brain tumor surgery.

## Simple Summary

Patients with gliomas show an increased risk of spontaneous hemorrhages throughout the disease. Simultaneously, the number of patients taking acetylsalicylic acid (ASA) for primary and secondary prophylaxis is rising in daily clinical practice. In the current study, we evaluate the risks associated with continuing ASA use perioperatively, focusing on hemorrhage and potential thromboembolic events that may arise from discontinuing ASA, particularly in multimorbid patients undergoing glioma surgery. The current findings show that the perioperative use of ASA is not associated with a significantly increased rate of hemorrhagic complications in glioma surgery. Thus, in patients with high stroke or cardiovascular risk, ASA can be continued during elective brain tumor surgery.

## 1. Introduction

Gliomas account for about 30% of all primary brain tumors and 80% of malignant brain tumors, and they also cause the most deaths in patients with primary brain tumors [1]. Based on their microscopic appearance and molecular characteristics, gliomas are classified according to the WHO classification of central nervous system (CNS) tumors and are further subdivided into CNS WHO grades 1 to 4, ranging from low to high malignancy [2]. Advances in molecular diagnostics have enabled a deeper understanding of the genetic and molecular characteristics underlying gliomas, paving the way for more personalized treatment strategies. Targeted therapies present promising opportunities to enhance glioma treatment while minimizing side effects. Functional neurosurgical, minimally invasive techniques—particularly focused ultrasound surgery (FUS) and laser interstitial thermal therapy (LITT)—provide the potential for precise and controlled tumor ablation, reducing damage to surrounding healthy brain tissue and supporting improved patient recovery [3]. To subdivide groups of glioblastoma (GBM), which allows for a better understanding of tumor complexity for personalized treatment, one can use clustering algorithms. Advanced analytical methods can be integrated with neuroimaging approaches, such as diffusion imaging and MR spectroscopy, leading to the development of targeted therapeutic strategies for GBM [4].

Intracranial hemorrhage (ICH) can occur in patients with GBM even without anticoagulation therapy due to the presence of angiogenic factors such as vascular endothelial growth factor (VEGF) and matrix metalloproteinases, as well as abnormal blood vessels within the tumor [5,6]. Spontaneous ICH in GBM is estimated to occur at a rate between 6 and 13%. With therapeutic anticoagulation, the incidence of ICH can increase up to three-fold, although there are conflicting data on the extent of this heightened risk [6,7,8,9,10]. POH in neurosurgery is one of the most dangerous complications after craniotomies and is associated with significant morbidity and mortality [11,12,13]. Several factors can negatively influence POH, including disorders of various coagulation factors, peri- and postoperative hypertensive conditions, the type and location of intracranial pathology, and age [11,12,13,14].

Antiplatelet agents like ASA are widely used as anticoagulants, particularly for the primary or secondary prevention of cardiovascular disease [13,15]. In as early as 1979, Merriman et al. showed that using ASA is associated with neurosurgical hemorrhage [9]. In a 5-year retrospective study, Palmer et al. [16] demonstrated that using antiplatelet agents is one of the most common risk factors for developing postoperative hematomas in neurosurgery. ASA increases the risk of bleeding complications during surgery by a factor of 1.5 without increasing mortality [17]. The current guidelines of the European Society of Cardiology (ESC) generally recommend the continued perioperative administration of ASA as part of secondary prevention [18]. In intracranial operations, however, even minor bleeds can lead to high morbidity, making it necessary to discontinue ASA [16,19]. Because ASA increases the risk of bleeding complications during neurosurgical procedures, it has been postulated that the administration of ASA should be paused preoperatively in order to rule out an anti-aggregatory effect [16,20]. The administration of the substance must be paused for seven days before surgery [17]. If ASA is part of primary prophylaxis, treatment can be interrupted perioperatively [18].

Gliomas also confer an elevated risk for the development of venous thromboembolism (VTE) [6,21,22]. Patients with GBM are estimated to have a 15% to 30% chance of developing deep vein thrombosis (DVT) or pulmonary embolism (PE) during their illness [6,23,24,25,26]. Although anticoagulants and platelet aggregation inhibitors are frequently used, data on perioperative management, particularly the preoperative discontinuation or bridging of the medication, are limited. Ultimately, the risk of thrombosis must be weighed individually against an increased tendency toward bleeding [27].

In this study, we examined the risk of POH, especially in predisposed patients after cranial glioma surgery, depending on the histological/cytological grading and location of the tumor, under the influence of ASA taken postoperatively and during the hospital stay. We also assessed the probability of postoperative thromboembolic events following glioma resection, particularly in the subgroup of patients with heightened cardiovascular risk, with and without the influence of ASA.

## 2. Materials and Methods

**Figure 1:**
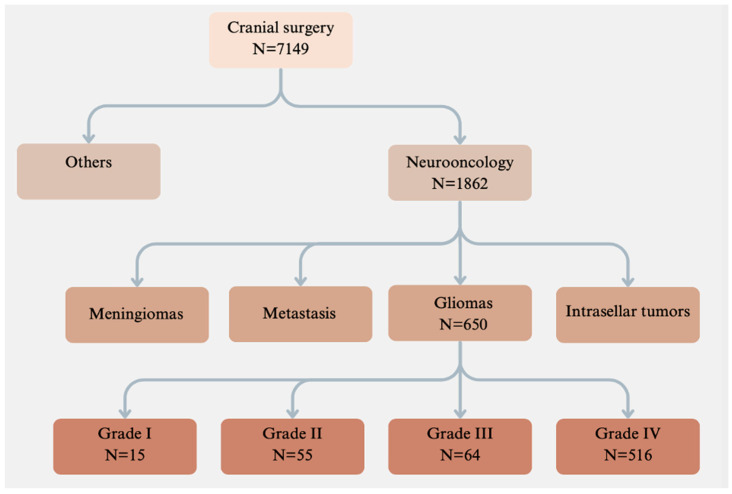
Of the 7149 patients who underwent surgery at our neurosurgical institute over the past decade, 650 underwent surgery for gliomas. The gliomas were classified according to the WHO classification system.

The medical records and radiological images of patients who underwent craniocerebral surgery at our institution between 2008 and 2018 were analyzed retrospectively. During this period, a total of 7149 patients received craniocerebral surgery for intracranial tumors, of which 650 surgeries were performed for gliomas. Figure 1 illustrates a flowchart of patients who underwent glioma surgery.

This retrospective review encompassed various patient data, including age, sex, blood group, body mass index (BMI), perioperative ASA administration, hypertension, diabetes, smoking history, cardiovascular disease, kidney disease, chronic inflammation, recurrence of glioma surgery, laboratory parameters, length of hospital stay, histological grading, tumor location, duration of surgery, blood loss, and postoperative complications. Complications during hospitalization were classified according to the system developed by Ibanez et al. as follows:

- Grade I: non-life-threatening abnormalities that deviated from the usual postoperative course and were managed without invasive procedures.
- Grade II: complications that required invasive interventions, including surgical, endoscopic, or endovascular procedures.
- Grade III: life-threatening adverse events that necessitated treatment in an intensive care unit, further divided into IIIa (complications involving single-organ dysfunction) and IIIb (complications involving multiple-organ dysfunction).
- Grade IV: complications resulting in death.

Unless contraindicated, preoperative and postoperative imaging with contrast-enhanced MRI was performed. Steroids were administered preoperatively when tumor edema or significant space-occupying effects were present. During the surgery, surgeons had access to intraoperative ultrasound, electrophysiological monitoring, and, if necessary, frameless neuronavigation. Tumor characteristics were assessed, including histologic grading, tumor size and location, the history of repeat surgeries, and the extent of resection. The operative parameters considered included blood loss, the duration of the surgery, and the extent of resection.

The Karnofsky Performance Scale (KPS) was used to assess patient status before surgery, while the KPS and the Glasgow Outcome Scale (GOS) were employed postoperatively.

Exclusion criteria included being under 18 years of age, pregnancy, and the use of other antiplatelet agents such as Clopidogrel or anticoagulants like Marcumar. To statistically evaluate the potential impact of ASA on postoperative hemorrhage, Fisher’s exact and Mann–Whitney U tests were applied. Patients were classified into two groups:

- No ASA impact: patients with no history of ASA use and/or who had not taken ASA for at least 7 days before surgery.
- ASA impact: patients who had stopped ASA intake less than 7 days before surgery or had not stopped it at all.

### 2.1. Intracranial Hemorrhage

All postoperative radiological findings were carefully examined for evidence of hemorrhage. The volume of blood was quantified by radiology specialists.

Hemorrhages were classified into the following categories:

- Hemorrhage within the tumor cavity.
- Intracerebral hemorrhage.
- Subarachnoid hemorrhage.
- Subdural hemorrhage

**Figure 2:**
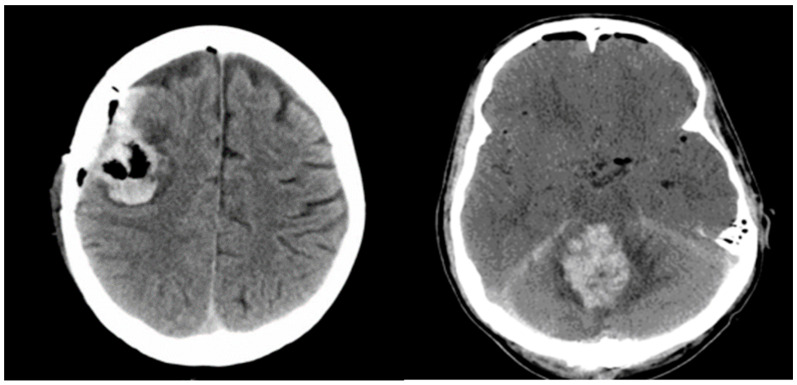
Illustrates two cases of postoperative hemorrhage that required reoperation.

Significant postoperative hemorrhage was defined as bleeding that led to notable neurological symptoms, such as increasing intracranial pressure and space-occupying effects requiring surgical intervention. Symptomatic neurological deficits included focal neurological deficits, headaches, vomiting/nausea, or changes in cognitive function. Figure 2 illustrates two cases of postoperative hemorrhage that required reoperation.

### 2.2. Statistical Analysis

Categorical variables are presented as counts and percentages, while continuous variables are expressed as medians with interquartile ranges (IQRs), as all continuous variables in this study were non-normally distributed, a fact confirmed by the Kolmogorov–Smirnov test. The association between categorical variables and POH was analyzed using the χ^2^ or Fisher’s exact test when cell counts were fewer than 5. Differences in continuous variables between patients with and without POH were compared using the Wilcoxon–Mann–Whitney test. Variables found to be significant in the univariate analysis were included as covariates in the multivariate analysis, which was conducted using logistic regression. For variables showing a substantial deviation from a normal distribution, a logarithmic transformation was applied. All statistical analyses were performed using the SAS University Edition software package version 9.4 (SAS Institute, Inc., Cary, NC, USA) and SPSS for Windows version 18.0 (SPSS, Inc., Chicago, IL, USA). Two-sided *p*-values < 0.05 were considered statistically significant.

## 3. Results

During the period mentioned above, 650 patients with different types of gliomas underwent surgery. The no ASA impact group included 577 patients (88.8%), and the ASA impact group included 73 patients (11.2%).

A total of 93 patients were on long-term ASA medication prior to surgery. Of these, 20 patients discontinued ASA more than 7 days before the operation. In 73 cases, ASA was stopped less than 7 days before the procedure due to concerns related to stroke and cardiovascular disease, but the elective surgery was still conducted.

POH primarily presented in two forms: intraparenchymal hemorrhage (n = 12) and hemorrhage in the resection cavity (n = 31) following tumor removal. Other forms of POH, such as epidural hematomas (n = 3), subdural hematomas (n = 3), or subarachnoid hemorrhages (n = 1), were less common than the two main types mentioned above.

### 3.1. Incidence of Postoperative Intracranial Hemorrhage

**Figure 3:**
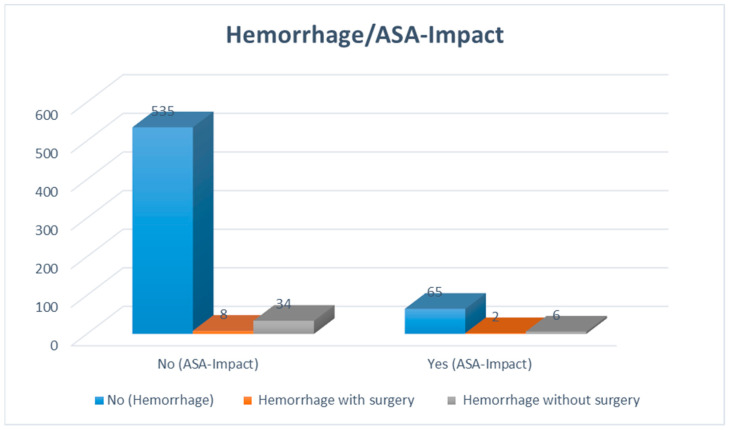
Out of 650 surgical patients with gliomas, 10 patients had a postoperative hemorrhage that required revision surgery due to clinical deterioration and the space-occupying effect of the hemorrhage.

During the specified period, a total of 50 patients with POH were identified through CCT and/or MRI scans, representing approximately 7.7% of the 650 patients. Only 10 patients (1.5%) with POH experienced clinical deterioration or had space-occupying hemorrhages requiring reoperation (Table 1 and Figure 3). Bivariate statistical analysis showed no significant differences (*p* = 0.098); in the ASA impact group, 2.7% developed POH, compared to 1.3% in the no ASA impact group. This indicates a trend toward an approximately 2-fold higher incidence of POH in the ASA impact group compared to the group without ASA.

In relation to demographic characteristics and additional data, factors such as sex (*p* = 0.755), smoking status (*p* = 0.697), age at the time of surgery (*p* = 0.952), BMI (*p* = 0.262), and the American Society of Anesthesiologists classification (*p* = 0.088) showed no significant impact on postoperative hemorrhage.

### 3.2. Comorbidities

Preoperative conditions like diabetes (*p* = 0.391), cardiovascular disease (*p* = 0.139), hypertension (*p* = 0.756), coagulopathy (*p* = 0.819), and chronic inflammation (*p* = 0.709) did not demonstrate a significant effect on the risk of postoperative hemorrhage.

### 3.3. Surgical Parameters

Neither the duration of surgery (*p* = 0.214) nor the intraoperative blood loss (*p* = 0.250) significantly influenced the risk of postoperative hemorrhage. However, postoperative KPS (*p* < 0.001) and GOS (*p* < 0.001) metrics were significant in patients from both groups following hemorrhage. Patients who experienced postoperative hemorrhage were in a worse overall condition after surgery, contributing to the significance of both parameters. Table 2 summarizes the factors influencing postoperative bleeding after glioma surgery. The tumor resection rate also showed no significant difference regarding postoperative hemorrhage between the two groups (*p* = 0.369).

### 3.4. Hemorrhage, Tumor Characteristics, and Laboratory Parameters

Tumor characteristics, including histopathological glioma type (*p* = 0.875), recurrent glioma (*p* = 0.735), glioma location (frontal, temporal, and skull base) (*p* = 0.688), and tumor side (*p* = 0.551), had no significant effect on the risk of postoperative hemorrhage in both patients with and without an ASA impact. Among the laboratory parameters, platelet count (*p* = 0.037) and C-reactive protein (CRP) levels (*p* = 0.013) were significantly associated with the development of POH. Table 3 summarizes the relevant preoperative laboratory parameters, histological tumor characteristics, and their impact on POH.

### 3.5. Patients Taking ASA

The average age of the patients in the ASA impact group was significantly higher at the time of surgery than that in the no ASA impact group (*p* < 0.001). A total of 307 (47.2%) patients were female, and 343 (52.8%) were male, with no significant difference between the two groups (*p* = 0.381). The American Society of Anesthesiologists classification score was significantly higher in the ASA impact group (*p* < 0.001), indicating that these patients had a worse overall condition than those without ASA. However, BMI (*p* = 0.139) and smoking status (*p* = 0.571) did not correlate significantly in either group.

### 3.6. Comorbidities

Arterial hypertension (*p* < 0.01), cardiovascular disease (*p* < 0.01), dyslipoproteinemia (*p* < 0.01), and renal insufficiency (*p* = 0.06) significantly differed between patients with and without preoperative ASA use. Diabetes mellitus (*p* = 0.08) also showed a difference, though it was not statistically significant (Table 4).

### 3.7. VTE and ASA Intake

In the group with no ASA impact, 12 of the 577 patients (2.1%) developed pulmonary artery embolism (PE), while 3 of the 73 patients (4.1%) in the ASA impact group experienced this outcome (Table 5). No significant difference was observed between the two groups (*p* = 0.232). In our cohort, 14 patients suffered from deep venous thrombosis (DVT), with only 1 of them being on ASA medication (Table 6).

### 3.8. Complications According to Ibanez Classification

Table 7 presents the frequency of complications in both groups using the Ibanez classification. It showed no significant difference (*p* = 0.551). However, patients with Ibanez complications classified as IIIa/IIIb were more frequently associated with bleeding.

## 4. Discussion

Gliomas and meningiomas are among the most common primary brain tumors [2]. Advanced age is a known risk factor for glioma [1]; the only proven risk factor for the development of glioma is exposure to ionizing radiation [28]. Children who suffered from long-term cancer and were treated with brain radiation had an increased risk of brain tumors such as gliomas and meningiomas [29].

The incidence of spontaneous hemorrhages, especially in glioblastomas, is higher in patients on therapeutic anticoagulation and may even increase three-fold [6,7,8,9]. The administration of ASA as an anticoagulant is now widely established as essential for treating various diseases [15]. Postoperative hemorrhage after cranial surgery can have disastrous consequences for the patient [16]. The mortality rate for patients experiencing intracranial postoperative hemorrhage is estimated to be around 30% [16,30,31]. Additionally, studies have shown that postoperative hemorrhage is the leading cause of death following cranial surgery [13,30,32].

The proportion of clinically relevant POHs in our patient population was 1.5%, which is within the range reported in the literature (0.8–6.9%) [32,33]. In our study, we also included all patients who had minimal residual hemorrhage in the resection cavity without any clinical symptoms. The rate of POH varies greatly between studies, depending on the definition [13]. The frequency of hemorrhage in patients with clinical deterioration who required surgery due to bleeding was double in the ASA impact group, at 2.7%, compared to 1.3% in the no ASA impact group. However, there was no significant statistical difference. Our findings are consistent with studies investigating the relationship between ASA and postoperative hemorrhage [34,35,36]. In a recent study, Rahman et al. analyzed patients undergoing craniotomies for intracranial tumors, and they also found no significant differences in the occurrence of postoperative intracranial hemorrhage or thromboembolic systemic complications between those taking ASA and the control group [19].

Several studies on cranial surgery also did not find an increased risk of POH related to surgery per se [19,37,38,39]. In a recent study, we found that the risk of postoperative hemorrhage was higher with preoperative ASA intake for certain tumor entities such as meningiomas [40].

An analysis of patient demographics and additional data revealed no significant differences between the ASA and no ASA impact groups. Some studies found that patients with pre-existing medical conditions had a higher incidence of hemorrhage [9,41]. However, these studies primarily examined the frequency of bleeding in relation to Enoxaparin administration, not ASA.

The age of the patient was identified as a significant factor for postoperative hemorrhage among the demographic data in some studies [30,42].

In Li et al.’s study, platelet counts below 150,000/µL were associated with an increased risk of POH [43]. Chan et al. found that platelet counts below 100,000/µL significantly elevated the risk of POH, which was also the case in our study. Among the laboratory parameters, only the platelet count showed relevance. In a study by Wang et al., INR was identified as a significant laboratory parameter for predicting postoperative intracranial hemorrhage. However, in the present study, INR did not show the same significance level. Patients who experienced postoperative hemorrhage showed a marked deterioration in their overall condition, with significant declines in both GCS and KPS scores following the hemorrhage.

Tumor characteristics, such as the WHO grade, tumor recurrence, and tumor location, did not reveal any significant correlation with the risk of postoperative hemorrhage. This could be due to the small number of patients in our study. The analysis of pre-existing conditions and their impact on postoperative bleeding revealed no significant influence in either group in our study. Most studies have identified hypertension as a significant factor in the development of hemorrhage, while diabetes mellitus, cerebral amyloid angiopathy, and atherosclerosis were generally not found to be significant [13,32]. However, Basali et al. observed that patients with postoperative intracranial hemorrhage had a notably higher rate of intraoperative and pre-hemorrhage hypertension [14].

Deep vein thrombosis (DVT) is a frequent complication in hospitalized and immobilized patients and can lead to pulmonary embolism (PE), which significantly increases the mortality rates of patients [44]. Patients with brain tumors are at an even higher risk of venous thromboembolism (VTE) due to factors such as neurological dysfunction and hypercoagulability [44]. A cohort study of 2638 neurosurgical patients found that the majority of DVTs occurred within the first week following surgery. The use of early subcutaneous heparin was linked to a 43% reduction in DVT incidence without increasing the risk of bleeding at the surgical site [45].

It is estimated that approximately 15% to 30% of patients with glioblastoma will develop DVT or PE over the course of their illness [6]. The reported data on VTE in brain tumor patients after craniotomy show considerable variations. For instance, Chaichana et al. reported a VTE incidence of 3.0% in these patients [46]. In contrast, in another study in which asymptomatic patients were also examined for the presence of VTE after surgery, a VTE incidence of 13.7% was found [47]. It is important to highlight that in our study, only symptomatic patients were further evaluated for thrombosis following glioma surgery. All patients were provided with compression stockings and encouraged to mobilize as early as the first postoperative day. Patients with paresis, who were unable to fully mobilize, still received professional physiotherapy with bedside movement exercises following surgery. Patients with an ASA impact had a pulmonary embolism rate of 4.1%, compared to 2.1% in those without an ASA impact. It is important to note that the higher rate of embolism complications in the ASA impact group is likely due to pre-existing medical conditions rather than the ASA itself, which is probably why these patients were using ASA for primary or secondary prophylaxis. For DVT, the incidence was 1.4% in the ASA impact group and 2.3% in the no ASA impact group. Despite the differing complication rates for DVT and PE, there was no other significant difference between the two groups.

## 5. Conclusions

Our study is limited by its retrospective design. Future prospective studies should validate these results through multiple assessments, including platelet function tests in patients on preoperative ASA. Additionally, some patients may be non-responders to ASA, and they should be excluded from the study following preoperative testing. A prospective, multicenter study with a larger patient population could overcome these limitations and provide greater statistical power by including more patients and additional subgroups.

The statistical analysis in this study did not show a clear influence of ASA on postoperative bleeding in patients with gliomas after surgery. However, there was a noticeable trend toward postoperative bleeding. Based on the available data, elective surgeries in patients with prior ASA intake should further minimize the risk of POH in ASA patients via early diagnostic imaging, and patients should be monitored more intensively.

## Figures and Tables

**Table 1:** Patients were classified based on the effects of ASA and hemorrhagic complications that required revision surgery. No significant differences were observed between the two groups.

				Hemorrhage with Surgery	No Hemorrhage	*p*-Value
				N (%)	N (%)	
ASA Impact	Yes No	∑ (%)	∑ 67 (11.0) 543 (89.0) 610	2 (20.0) 8 (80.0) 10 (100.0)	65 (10.8) 535 (89.2) 600 (100.0)	0.098

**Table 2:** Patients in both groups were categorized based on demographic data, comorbidities, and surgical parameters, which were analyzed to identify any correlations with hemorrhagic complications. None of these parameters demonstrated a significant impact on postoperative hemorrhage. However, the measurement results (KPS and GOS) showed a significant correlation with clinically relevant postoperative hemorrhage.

Parameters			Hemorrhage		
			No Hemorrhage N 601 (%), Mean # SD	Hemorrhage with Operation N 10 (%), Mean # SD	*p*-Value
Demographic data	Sex	Female Male	286 (47.6) 315 (52.4)	4 (40.0) 6 (60.0)	0.755
	Age		58.7	58.3	0.952
	BMI		27.7	29.6	0.262
	ASA classification	I II III–IV	35 (5.9) 355 (59.4) 210 (34.7)	0 (0.0) 5 (50.0) 5 (50.0)	0.088
	Smoker	Yes No	84 (23.3) 292 (77.7)	2 (25.5) 6 (75.5)	0.697
Surgical parameters	Duration of the operation, [min]		191.5	164.10	0.214
	Blood loss [mL]		330.8	430.0	0.250
	Duration of stay (days)		14.5	16.4	0.684
	Glasgow Coma Scale (GCS)		4.1	2.6	<0.001
	Karnofsky Performance Scale (KPS)		67.7	38	<0.001
Comorbidities	Hypertension	Yes No	317 (52.7) 284 (47.3)	6 (60.0) 4 (40.0)	0.756
	Diabetes	Type 1/2 No	104 (17.3) 497 (82.7)	3 (30.0) 7 (70.0)	0.391
	Coagulopathy	Yes No	12 (2.0) 589 (98.0)	0 (00.0) 10 (100.0)	0.819
	Cardiovascular	Yes No	79 (13.1) 522 (86.9)	3 (30.0) 7 (70.0)	0.139
	Chronic inflammation	Yes No	26 (4.3) 575 (95.7)	0 (0.0) 10 (100.0)	0.709

**Table 3:** Laboratory parameters and tumor characteristics were analyzed in relation to postoperative hemorrhage. Among them, the thrombin count demonstrated a significant impact on the risk of postoperative hemorrhage. None of the other parameters were relevant risk factors for developing postoperative intracerebral hemorrhage.

Parameters			Hemorrhage		
			No Hemorrhage N 601 (%), Mean ± SD	Hemorrhage with Operation N 10 (%), Mean ± SD	*p*-Value
Laboratory parameters	INR		0.98	0.98	0.957
	Platelets [10*9/L]		261	212	0.037
	C-reactive protein [mg/L]		6.5	2.2	0.013
	Leukocytes [Gpt/L]		9.7	12.0	0.174
Tumor characteristics (gliomas)	Recurrence	yes, no	165 (27.6) 432 (72.4)	2 (20.0) 8 (80.0)	0.735
	Localization	frontal, temporal parietal, occipital, cerebellar, intra/suprasellar, skull base, CPA	202 (33.6) 153 (25.5) 151 (25.1) 61 (10.1) 12 (2.0) 1 (0.2) 19 (3.2) 1 (0.2)	2 (20.0) 5 (50.0) 2 (20.0) 1 (10.0) 0 (0.0) 0 (0.0) 0 (0.0) 0 (0.0)	0.688
	Side	left, middle, right	280 (46.6) 35 (5.8) 286 (47.6)	5 (50.0) 0 (0.00) 5 (50.0)	0.551
	WHO grade	I II III IV	15 (2.5) 49 (8.2) 61 (10.1) 476 (79.2)	0 (0.0) 1 (10.0) 1 (10.0) 8 (80.0)	0.875

**Table 4:** Demographic data of the ASA groups revealed that patients in the ASA impact group were older than those in the no ASA impact group; had a worse ASA classification score; and more frequently suffered from comorbidities such as arterial hypertension, cardiovascular disease, and diabetes.

Parameters			Preoperative ASA Use		
			No ASA Impact N 557 (%), Mean ± SD	ASA Impact N 73 (%), Mean ± SD	*p*-Value
Demographic data	Sex	Female Male	269 (46.6) 308 (53.4)	38 (52.1) 35 (47.9)	0.381
	Age		57.6	69.3	**<0.001**
	BMI		27.5	28.4	0.139
	ASA classification	I II III–IV	36 (6.3) 346 (60.3) 192 (33.5)	0 (0.0) 34 (46.6) 39 (53.4)	**<0.001**
	Smoker	Yes No	119 (21.2) 442 (78.8)	13 (18.3) 58 (81.7)	0.571
Operational factors and duration of stay	Duration of the operation [min]		190	203.5	0.185
	Blood loss [mL]		338	386	0.134
	Duration of stay (days)		14.6	15.3	0.134
Comorbidities	Hypertension	Yes No	291 (49.6) 286 (47.3)	61(83.6) 12 (16.4)	**<0.001**
	Dyslipoproteinemia	Yes No	46 (8.0) 531 (92.0)	17 (23.3) 56 (66.7)	**<0.01**
	Renal failure	Yes No	23 (4.0) 554 (96.0)	9 (12.3) 64 (87.7)	**0.06**
	Diabetes	Type 1/2 No	94 (16.3) 483 (83.7)	21 (28.8) 52 (71.2)	**0.08**
	Coagulopathy	Yes No	11 (1.9) 566 (98.1)	2 (2.7) 71 (97.3)	0.520
	Cardiovascular disease	Yes No	59 (10.2) 518 (89.8)	29 (39.7) 44 (60.3)	**<0.001**
	Chronic inflammation	Yes No	26 (4.5) 551 (95.5)	4 (5.5) 69 (94.5)	0.765

**Table 5:** No significant difference was found between the ASA and non-ASA groups regarding post-surgical PE development.

				PE	No PE	*p*-Value
				N (%)	N (%)	
**ASA Impact**	**Yes** **No**	∑ (%)	∑ 73 (100%) 577 (100%) 650	3 (4.1%) 12 (2.1%) 15 (2.3%)	70 (95.9%) 565 (97.9%) 635 (97.7%)	0.232

**Table 6:** No significant difference was found between the two groups with regard to the development of DVT.

			DVT	No DVT	*p*-Value
			N (%)	N (%)	
**Yes** **No**	∑ (%)	∑ 73 (100%) 577 (100%) 650	1 (1.4%) 13 (2.3%) 14 (2.2%)	72 (98.6%) 564 (97.7%) 636 (97.8%)	0.0504

**Table 7:** There was no substantial difference between the groups with or without ASA. However, a significant correlation between severe complications was observed, especially in patients with postoperative hemorrhage (*p* = 0.001).

Surgical Complications According to Ibanez’s Classification * Hemorrhage					
			ASA Perioperative		Total
			No ASA Impact	ASA Impact	
Surgical complications	0	N (%)	402 (69.7%)	51 (69.9%)	453 (69.7%)
END	Ia/Ib	N (%)	115 (19.9%)	18 (24.7%)	133 (20.5%)
CFS leak/wound infection	IIa/IIb	N (%)	29 (5.0%)	1 (1.4%)	30 (4.6%)
Hemorrhage	IIIa/IIIb	N (%)	30 (5.2%)	3 (4.1%)	33 (5.1%)
Death	IV	N (%)	1 (0.2%)	0 (0.0%)	1 (0.2%)
Total		N (%)	577 (100%)	73 (100%)	650 (100%)

## Data Availability

The datasets obtained and analyzed during the current study are available from the corresponding author upon reasonable request.
